# Imaging of α2C-adrenoceptors in the living brain: a method to monitor noradrenaline release?

**DOI:** 10.1186/2193-1801-4-S1-L20

**Published:** 2015-06-12

**Authors:** Mika Scheinin, Jussi Lehto, Jarkko Johansson, Pauliina Luoto, Eveliina Arponen, Lauri Vuorilehto, Harry Scheinin, Juha Rouru

**Affiliations:** University of Turku, Finland; Turku PET Centre, TYKS, Finland; Orion Corporation, Finland

**Keywords:** Adrenoceptor, noradrenaline, positron emission tomography

## Objectives

The PET tracer [11C]ORM-13070 was recently validated for receptor occupancy analysis of brain α2C-adrenoceptors, and PET experiments in monkeys and humans indicated that tracer uptake into the caudate and putamen was reduced by interventions that increased synaptic noradrenaline concentrations in the brain [1–3]. This study aimed to confirm the sensitivity of [11C]ORM-13070 binding to increased levels of synaptic noradrenaline.

## Methods

PET imaging of the brain was performed with a 3D High Resolution Research Tomograph. Eight subjects underwent a control [11C]ORM-13070 PET scan and two PET scans after two different noradrenaline challenges, i.e. a sub-anaesthetic infusion of ketamine and oral intake of atomoxetine combined with cold stimulation. Tracer uptake in the caudate nucleus and putamen was described with AUC values in scan time windows of 10-20 min and 5-30 min post injection, and quantified with the ratio method. Voxel-based analysis was performed with average B/F images. Both challenges caused small but statistically significant (10-20%, p<0.05) reductions in tracer uptake in both target regions. Voxel-based analysis revealed significant clusters in the dorsal putamen with both challenges. Ketamine was associated with significant elevations in circulating noradrenaline and adrenaline levels, while the atomoxetine + cold treatment was not. Strong experimental support was gained for the feasibility of [11C]ORM-13070 PET imaging of brain noradrenergic neurotransmission.
